# Isolation and Genomic Characterization of a Lytic Bacteriophage Against Multidrug-Resistant *E. coli*

**DOI:** 10.3390/v18050532

**Published:** 2026-04-30

**Authors:** Ramesh Kumpatla, Vinaya Kruthi Vitala, Arunasree M. Kalle

**Affiliations:** Department of Animal Biology, School of Life Sciences, University of Hyderabad, Hyderabad 500046, Telangana, India; ramesh.genetics6@gmail.com (R.K.); 21laph01@uohyd.ac.in (V.K.V.)

**Keywords:** bacteriophages, multidrug-resistant *E. coli*, lytic phages, biofilm degradation, whole genome sequencing, host-range specificity

## Abstract

Antimicrobial resistance (AMR) has become a major concern in the treatment of bacterial infections, and bacteriophage therapy has emerged as a promising alternative to antibiotics. Bacteriophages are highly specific to their bacterial hosts; hence, isolating phages indigenous to a specific region offers a significant advantage against various pathogen strains. We have isolated a cocktail of bacteriophages against pathogenic *E. coli* from sewage water at a primary healthcare centre. Characterisation of the isolated phages demonstrated their stability across a broad pH and temperature range, strong lytic activity, and effective biofilm degradation, with no cross-reactivity with *Staphylococcus aureus* (*S. aureus*). Genomic analysis and phylogenetic studies indicated that the largest phage (by genome size) in the cocktail belongs to the genus *Vequintavirus* (myoviruses, rV5-like phages), and its genome sequence has been deposited in NCBI (Accession ID: PX741096). The phage genome was linear, with headful (PAC) packaging, encoded lysis proteins, and lacked antibiotic-resistant or major lysogeny-associated genes, collectively suggesting a lytic lifestyle. These findings emphasize the therapeutic potential of rV5-like phages and underscore the critical need to establish phage banks in India to improve disease management.

## 1. Introduction

*Escherichia coli* (*E. coli*) is a Gram-negative, facultative anaerobic bacterium belonging to the family *Enterobacteriaceae*, mainly inhabiting the intestines of warm-blooded animals [1]. While most of the *E. coli* strains are harmless, certain strains are pathogenic. They can cause either intestinal infections, such as those caused by enteropathogenic (EPEC), enterotoxigenic (ETEC), Shiga toxin-producing *E. coli* (STEC), or extra-intestinal infections such as urinary tract infections and sepsis [2,3].

Antimicrobial resistance (AMR) poses a severe challenge to the treatment of bacterial infections [4]. AMR was directly responsible for 1.27 million deaths globally in 2019 and may rise to 10 million deaths per year by 2050 without proper measures [5]. Several mechanisms facilitate adaptive antibiotic resistance, including epigenetic inheritance, mutations, horizontal gene transfer (HGT), and efflux pumps [6]. Biofilm formation offers a protective barrier that restricts antibiotic penetration and promotes bacterial persistence and AMR [7]. The role of biofilms in antibiotic resistance is very complex, with bacteria exhibiting a 10 to 1000-fold increase in resistance compared to their planktonic state [8]. Biofilms perpetuate AMR mainly through three mechanisms: (1) resistance at the biofilm surface (ECM), (2) metabolic heterogeneity (anaerobic environment), and (3) survival of persister cells [8]. The emergence of multidrug-resistant (MDR), extensively drug-resistant (XDR), and pan-drug-resistant (PDR) strains has limited the therapeutic efficacy of antibiotics, highlighting the urgent need to explore alternative treatment options.

One promising alternative strategy for the prophylaxis and control of bacterial infections is phage therapy, which offers several benefits over antibiotics, including host specificity, self-amplification, biofilm degradation, and minimal human toxicity [9]. With technical advancements like next-generation sequencing (NGS) and electron microscopy, phage therapy has renewed interest as a viable clinical treatment option. Several studies have already demonstrated the effectiveness of phage therapy in treating drug-resistant bacterial infections [10,11]. Phage therapy has been a part of health care in some European countries, and it has regained momentum globally, including in India, the USA, China, etc. [12].

Due to the extreme diversity of phages and the continuous evolution of bacterial hosts, it is necessary to isolate and characterize phages specific to indigenous clinical pathogens to establish and develop local reference phage banks [13]. In the present study, we isolated and characterized phages collected from sewage at a local primary health care centre against pathogenic *E. coli* strains.

## 2. Materials and Methods

### 2.1. Sample Collection

250 mL of untreated slow-flowing sewage water was collected in a sterile glass bottle from the on-campus Primary Health Care Centre at the University of Hyderabad, Hyderabad, TS, India, on 22 May 2020. The bottle was then transported on ice to our laboratory and processed immediately [14].

### 2.2. Bacterial Strains and Culture Conditions

A total of 10 *E. coli* strains, 8 pathogenic clinical isolates and 2 non-pathogenic laboratory strains (*E. coli* K-12 and DH5α), and 10 *S. aureus* clinical strains were used in the study. Among them, the K-12 strain was used as the host strain for isolation, purification, and lyophilization. The bacterial isolates were collected from the Asian Institute of Gastroenterology, Hyderabad, India, during 2012 to 2013, and the glycerol stocks were maintained at −80 °C. Bacterial identification and antibiotic profiling were performed by the hospital microbiologist using an automated API system. However, strains were not characterized genotypically. The clinical strains were collected after obtaining approvals from the Institutional Ethical Committee (IEC No: UH/IEC/2015/85) and Institutional Biosafety Committee (IBSC-AMK-N-80) of the School of Life Sciences, University of Hyderabad, India. All clinical strains used in this study were classified as multidrug-resistant based on their antibiogram profiles (Supplementary Table S1. xlsx). The bacteria, both *E. coli* and *S. aureus*, were recovered from the glycerol stock by streaking onto an LB agar (HiMedia, Thane, India) plate and grown overnight at 37 °C. A single isolated colony was resuspended in LB broth (HiMedia, India) and grown at 37 °C to an adjusted 0.5 McFarland for the spot/plaque assay.

### 2.3. Isolation

Bacteriophages present in the collected sample were enriched using a previously established protocol to remove bacteria and debris [14]. In short, sewage samples were clarified by centrifugation (Eppendorf, Hamburg, Germany) at 9000× *g* for 10 min at 4 °C, followed by filtration of the supernatant with a 0.2 µm PVDF syringe filter (Millipore, Darmstadt, Germany). A 50 mL aliquot of this filtrate was mixed with 50 mL of 2× LB in a 500 mL flask and inoculated with 50 µL of early log-phase (3 h) K-12 (host) bacteria. After incubation at 37 °C and 150 rpm for about 18–24 h, the culture was centrifuged at 9000× *g* for 10 min at 4 °C, and the supernatant was filtered through a 0.2 µm PVDF syringe filter.

### 2.4. Plaque Assay

A double-layer agar plaque assay was performed to verify the presence and activity of phages. 200 µL of early log-phase *E. coli* K-12 bacterial culture was mixed with 200 µL of each diluted and undiluted phage lysate and incubated for 10–15 min at room temperature. This 400 µL mixture was added to 4 mL of soft agar, poured onto Petri plates (Tarsons, Kolkata, India) with hardened bottoms (LB) agar, left to dry, and later incubated for 18–24 h at 37 °C [15]. Plaques were counted, and the phage titer was estimated as plaque-forming units (PFU/mL) and calculated by the following formula [15]:

PFU/mL = (Number of plaques × Dilution factor)/Volume plated (mL).

### 2.5. Dia-Filtration or Cross-Flow Filtration for the Concentration of Phages

Approximately 900–1000 mL of 0.2 µm-filtered phage lysate obtained after enrichment in 2× LB broth with the K-12 strain was concentrated using a Sartorius Vivaflow 50R cross-flow filtration cassette (Sartorius, Göttingen, Germany) according to the established protocol [16]. The lysate was recirculated until the volume was reduced, and the final concentrated lysate was collected in two fractions: 40 mL and 30 mL.

### 2.6. Lyophilization of Purified Phage Lysate

The concentrated phage lysate was lyophilized using a 0.5 M sterile sucrose solution at a 1:3 ratio and freeze-dried using Labconco FreeZone Freeze Dryer (Labconco, Kansas City, MO, USA) [17]. The samples were precooled at −20 °C, and sample vials were loaded onto lyophilizer shelves. The shelves were cooled to −30 °C (−1 °C/min) and maintained for 90 min. Primary drying at −30 °C for 12 h at 100 millitorr, and during the secondary drying, the temperature was increased to 25 °C (0.1 °C/min) for 10 h at 100 millitorr.

### 2.7. Characterization of Phages

#### 2.7.1. Thermal Stability

The thermal stability of purified phages was examined by pre-incubating the phage suspension in LB broth (100 μL lysate + 900 μL LB broth) at various temperatures (4 °C, 16 °C, 25 °C, 37 °C, 46 °C, 60.9 °C, 70 °C, and 90 °C) for an hour. After incubation, the phage suspensions were immediately cooled to 4 °C for 5 min and spotted onto a lawn of host bacteria using the double-layer agar method.

#### 2.7.2. pH Stability

The pH stability of phages was evaluated by pre-incubating the phage suspensions in 0.1 M Phosphoric acid buffer (pH 2.1), sodium citrate buffer (pH 4.4), sodium phosphate buffer (pH 7), and Tris–HCl (pH 9 and 10.2) (Sigma, Kawasaki-shi, Japan) at 25 °C for 1 h followed by dilution in LB broth (100 μL lysate + 900 μL LB broth). The surviving phages were spotted on the lawn of host bacterial cells using the double-layer agar method. Three technical replicates were used for each pH level.

#### 2.7.3. Phage Morphology

The purified phage particles were transferred to the surface of a formvar carbon film (on a carbon film copper grid) (Sigma, Japan), negatively stained with UranyLess solution (Delta Microscopies, Mauressac, France) [18]. The stained specimens were dried using filter paper and observed using a JEM-1400 transmission electron microscope (TEM) (JEOL, Tokyo, Japan) at 12,000× magnification.

#### 2.7.4. Host Range Specificity

The purified and lyophilized phage lysate, in biological replicates, was tested for host specificity using human clinical isolates of *E. coli* and *S. aureus*, following the standard spot assay protocol. 200 µL of early log-phase host bacterial culture was mixed with 4 mL of soft agar in a test tube, gently swirled, and poured onto Petri plates with hardened bottoms (LB) agar, then left to solidify. 5–10 µL of undiluted and serially diluted phage lysate was uniformly spotted on the agar plate, allowed to dry for 15 min, and incubated for 18–24 h at 37 °C [14,15].

#### 2.7.5. Quantification of Biofilm Formation and Degradation

To further assess the therapeutic potential of isolated phages, we quantified their biofilm-degrading capacity in clinical isolates of *E. coli*. Therefore, first, the biofilm formation by the clinical strains was assessed. Different clinical *E. coli* strains were initially grown at 37 °C under shaking for 2 h. Then the culture of each strain was diluted (1:10) in fresh LB broth, and 200 µL of the diluted culture was added in triplicate to a 96-well microplate, while sterile LB served as a control. Then, culture plates were incubated for 24, 48, and 72 h for biofilm formation. After incubation, planktonic cells were removed by washing wells twice with 250 µL of PBS. The remaining adherent cells were stained with 250 µL of 0.1% crystal violet (CV) (HiMedia, India) at room temperature for 30 min, then washed 4 times with distilled water and air-dried for 1 h. Later, the bound CV was solubilised with 200 µL of acetone-ethanol (20:80) at room temperature and quantified by measuring the optical density at 595 nm (OD_595_) [19]. The relative biofilm mass was calculated by normalising the absorbance to that of the control at the corresponding time points (24, 48, and 72 h), which was set to 100% [19].

Next, *E. coli* biofilms were treated with phages to assess the biofilm degradation. *E. coli* cultures were incubated separately for 24, 48, and 72 h. After the initial incubation, the wells were washed twice with PBS, and 20 µL of phage solution in 180 µL of fresh LB broth was added. The plates were incubated for 24 h at 37 °C. After incubation, cells were stained with CV as described above, and the OD at 595 nm was determined. The relative biofilm mass was calculated by normalising the absorbance to that of the untreated control at the corresponding time points (24, 48, and 72 h), which was set to 100% [19].

### 2.8. Bacteriophage Genome Sequencing and Analysis

#### 2.8.1. Genomic DNA Isolation

Isolation of genomic DNA was carried out by pooling the 3 vials of lyophilized phages, each containing 10^4^ PFU/mL, followed by resuspension in 4 mL of saline magnesium (SM) buffer (50 mM Tris-HCl (pH 7.5), 100 mM NaCl, 10 mM MgSO_4_, 0.01% (*w*/*v*) gelatin) (Sigma, Japan). The phage suspension was placed on the rocker (Tarsons, India) for 1 to 3 h to isolate the top layer. Then, the SM buffer containing phages was centrifuged at 9000× *g* for 10 min at 4 °C, and the supernatant was filtered through 0.2 μm syringe filters to remove bacterial debris and media components. The phage pellet was precipitated using PEG8000 (Sigma, Japan) and NaCl, followed by high-speed centrifugation as described previously [20]. DNA was extracted from the phage pellet using the DNeasy^®^ Blood and Tissue Kit (Qiagen, Hilden, Germany) as per the manufacturer’s protocol.

#### 2.8.2. Genome Sequencing and Analysis

Phage DNA sequencing was performed on three replicates (phage lysate) using an Ion Torrent S5 platform (Thermo Fisher Scientific, Waltham, MA, USA) with an Ion 540 chip. Library preparation was performed using the Ion S5 AmpliSeq Library Kit Plus (SKU: 4488990) and Ion Code Adaptors 0101-0196 (SKU: A29747) according to the manufacturer’s protocol. The raw data were obtained and quality assessed using FASTQC version 0.12.1 [21]. Then, single-end reads were trimmed for quality and adaptor removal using Trimmomatic version 0.39 [22]. The de novo assembly of the genome was performed using SPAdes version 3.15.54 with Ion Torrent-specific parameters (–careful, –iontorrent) [23]. The quality of the genome assembly was assessed using Quast v5.2.0 [24].

#### 2.8.3. Identification of the Phage Regions

For the initial screening, the metagenome-like assemblies were analysed using the PHASTEST Version 3.0 (PHAge Search Tool with Enhanced Sequence Translation) web server [25]. A completeness score was calculated for each region based on its size, number of genes, and cornerstone genes that are essential for phage structure, DNA regulation, and lysis. Phage regions identified in the assemblies were classified into intact (score > 90), questionable (score 70–90), and incomplete (score < 70). Only those that were identified as intact were included in the study. To strengthen this prediction and reduce bias in prophage-prediction tools, the phage regions identified here were further confirmed using VIBRANT version 1.2.1, which predicts high-quality phages, infers their lifestyles and assesses genome completeness [26]. For an overall taxonomic classification, lifestyle prediction and host detection of the phage regions identified by VIBRANT across the three replicates, PhaBox was used [27]. Based on the above tools, phages that were consistently detected across the three assemblies were compared through Nucleotide BLAST v2.13.0 (Basic Local Alignment Search Tool) [28], and those with coverage > 80% and identity > 90% were considered to be the same phage and a representative of the dominant phage in the cocktail. The dominant phage was selected for further characterization. CheckV was used to assess the completeness and contamination of the phage genome [29]. Additionally, PhageTerm was used to analyse the genome packaging mechanism and to determine if the genome was linear or circular [30].

#### 2.8.4. Annotation of Phages

PHASTEST predicted the CDS, which were then manually curated using HMMER v3.3.2 (against the Pfam database) to identify related protein domain families with an E-value of 1 × 10^−5^ [31]. Then, additionally, eggNOG-mapper v2.1.12 was used to functionally annotate the CDS using orthology predictions [32].

For consistency, all annotations were compared, and mismatches were further investigated using Protein BLAST against the NCBI database. The annotated genome was visualised in PHASTEST using Genome Viewer 2.0. To establish the tail morphology group, the closest bacteriophage given by PHASTEST was searched in the viral-host database using its taxonomy ID [33]. A blast search of the terminase large subunit against the NCBI database was done to confirm the results. Additionally, RGI v6.0.3 (Resistance Gene Identifier) was used to find antibiotic-resistant genes in the phage genomes [34]. Anti-CRISPR proteins were also searched through CRISPRCasFinder v4.2.20 [35] and Acrfinder [36]. The presence of virulence genes in the phage genome assembly was detected using VirulenceFinder 2.0 [28,37,38].

#### 2.8.5. Phylogenetic Analysis of the BpE1AUH

The complete genome of the dominant phage, *Escherichia* phage BpE1AUH (BpE1AUH), was blasted against the NCBI database using BLASTn, and later, the intergenomic similarity between BpE1AUH and related phages was estimated using VIRIDIC [39].

The entire phylogenetic analysis was carried out by the VICTOR web service [40], a method for the genome-based phylogeny and classification of prokaryotic viruses [41]. All pairwise comparisons of the nucleotide sequences were conducted using the Genome-BLAST Distance Phylogeny (GBDP) method [42] under settings recommended for prokaryotic viruses [41]. The resulting intergenomic distances were used to infer a balanced minimum evolution tree with branch support via FASTME, including SPR postprocessing [43] for each of the formulas D0, D4 and D6, respectively. Branch support was inferred from 100 pseudo-bootstrap replicates each. Trees were rooted at the midpoint [44] and visualised with ggtree [45]. Taxon boundaries at the species, genus, and family levels were estimated using the OPTSIL program [46], the recommended clustering thresholds [41], and an F value (fraction of links required for cluster fusion) of 0.5 [47].

#### 2.8.6. Terminase Tree

The terminase large subunit protein sequence of the phage BpE1AUH was taken as a reference for BLASTp search against the NCBI non-redundant (nr) database. The top 20 hits, based on percentage identity, were selected for alignment using MAFFT v7.505 with the G-INS-i algorithm [48]. A maximum likelihood (ML) tree was constructed with IQ-TREE v2.1.4 [49]. This tree was later visualized with FigTree v1.4.4 using a circular layout and colour-coded genus-level annotations [50].

### 2.9. Statistical Analysis

The standard deviation of the data was obtained from triplicate experiments, and the statistical analysis was performed in GraphPad Prism 10.0.1 using a two-way ANOVA, with *p*-values indicating significance: * *p*-value < 0.05, ** *p*-value < 0.01, *** *p*-value < 0.001, and **** *p*-value < 0.0001.

## 3. Results

### 3.1. Isolation and Purification of E. coli-Specific Bacteriophages

**Figure 1:**
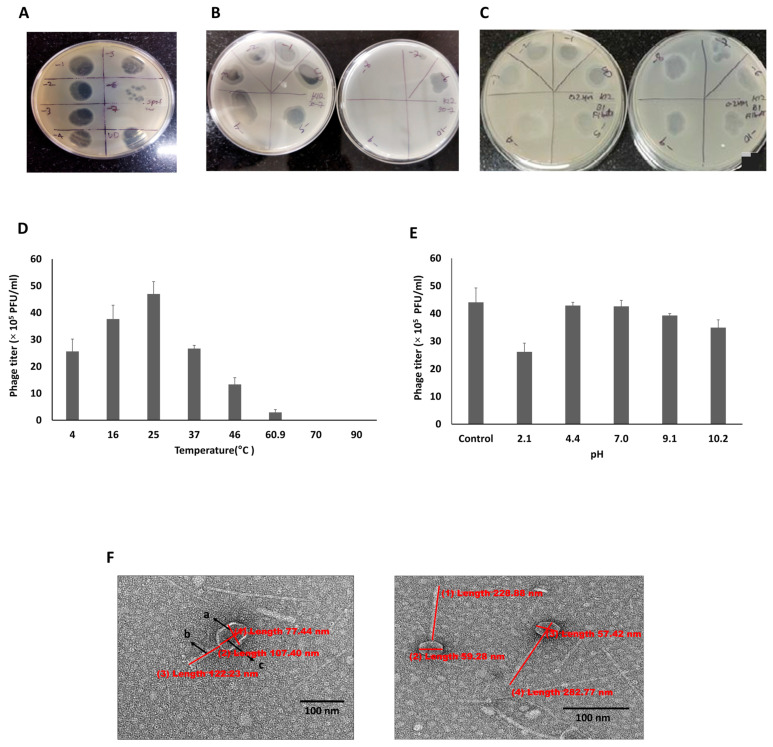
**Isolation, purification and characterization of *E. coli* specific bacteriophages.** (**A**) Plaque assay using undiluted and serially diluted phage lysate on *E. coli* K-12. (**B**) Spot assay for Vivaflow 50R purified phage lysate on *E. coli* K-12. (**C**) Phage assay for lyophilized phages. (**D**) Phage stability at different temperatures on *E. coli* K-12. (**E**) Phage stability at various pH values on *E. coli* K-12. (**F**) **Left** image: Bacteriophage morphology by TEM at 12,000× magnification with icosahedral capsid diameter (a) of 77.44 nm, length (c) of 107.40 nm, and 122.3 nm for contractile tail length (b). **Right** image: Phage cocktail with other phage types.

The collected sewage water sample was enriched as per the protocol mentioned in the methodology section. After incubation with K-12 cells and filtration, the lysate was tested for phage activity using a spot assay with undiluted and 10^−3^ diluted samples. When 10 μL of different dilutions were spotted, clear zones on a lawn of K-12 bacteria were observed at lower dilutions up to 10^−5^, while clear, isolated plaques were observed at 10^−6^ and 10^−7^. An average of 15 plaques per 10 μL spot was observed at 10^−6^ dilution (Figure 1A). Based on this, the phage titer of the undiluted lysate was determined to be 1.5 × 10^9^ PFU/mL.

A large-scale purification of the isolated phages was carried out from a one-litre culture using a Sartorius Vivaflow 50R cross-flow filtration system. The phage activity of the purified phages was determined using a spot assay. The purified phages showed higher activity up to a 10^−10^ dilution, with a titer of 3.3 × 10^5^ PFU/mL (Figure 1B). We further lyophilized the phages in 1 mL glass vials and determined their titer as 2.1 × 10^4^ PFU/mL (Figure 1C).

The temperature stability analysis showed that the activity of the lyophilized phages was retained between 4 °C and 46 °C, with the highest titer observed around 25 °C. There was a gradual reduction in the phage titer above 25 °C, and no activity was observed beyond 60.9 °C (Figure 1D).

Similarly, pH stability analysis revealed that phage activity was maintained across a broad pH range (2.1–10.2). Even though a slight decline in the titer was observed at highly acidic conditions (pH 2.1), the activity was retained across mild acidic, neutral, and alkaline pH conditions (Figure 1E).

Transmission electron microscopy of the lyophilized phages revealed morphological heterogeneity, within which the dominant phage relevant to the current study possessed an icosahedral capsid with a diameter of 77.4 nm and a tail with a contractile sheath measuring 122.23 nm (Figure 1F).

### 3.2. Purified Phages Were Specific to E. coli and Did Not Affect S. aureus

**Figure 2:**
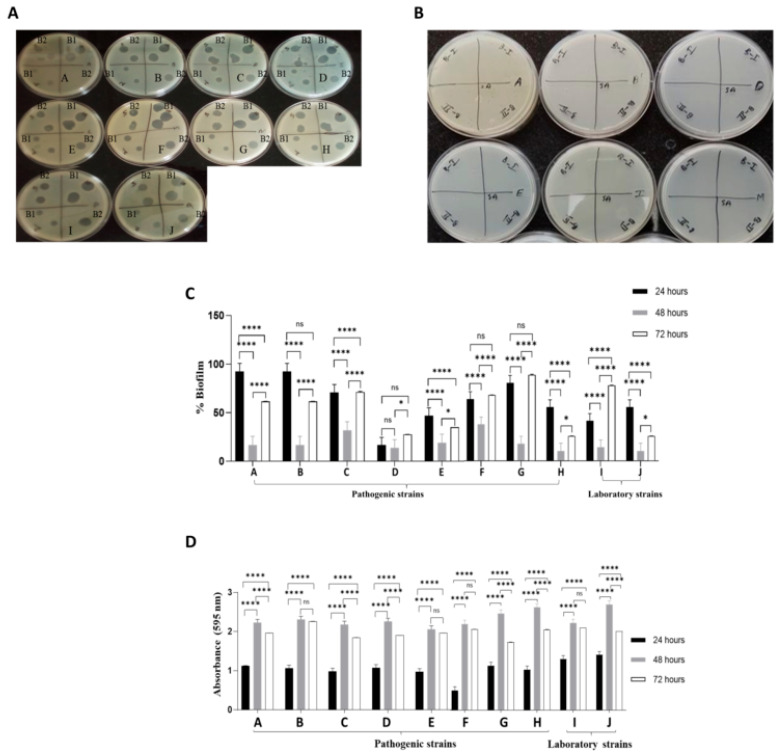
**Purified bacteriophages showed *E. coli* specificity and efficient biofilm degradation.** (**A**) Spot assay for host range specificity of phage lysate on pathogenic clinical *E. coli* strains (A–H) and non-pathogenic laboratory strains (I&J); B1 and B2 represent two biological replicates. (**B**) Spot assay for cross-reactivity of phage lysate on pathogenic clinical *S. aureus* strains (A, B, D, E, I, M); B-I and B-II represent two biological replicates. (**C**) Biofilm quantification of pathogenic and non-pathogenic *E. coli* Strains after 24, 48, and 72 h. * *p* ≤ 0.05, **** *p* ≤ 0.0001, ns *p* = 0.1423. (**D**) Biofilm degradation of pathogenic and non-pathogenic *E. coli* strains after 24, 48, and 72 h, followed by 24 h of exposure to phages. **** *p* ≤ 0.0001, ns *p* > 0.05.

The purified and lyophilized phages were evaluated for host specificity by spot assay using multidrug-resistant clinical strains of *E. coli* and *S. aureus* isolated from patient samples (Supplementary Table S1 (Supplementary Table S1. xlsx)). All tested *E. coli* strains were susceptible to the isolated phages (Figure 2A) and did not affect *S. aureus* strains (Figure 2B), suggesting that the phages were specific to *E. coli*.

Biofilms are a significant contributor to bacterial antibiotic resistance. To further evaluate the effect of isolated phages on biofilms formed by pathogenic *E. coli*, we first assessed biofilm formation by all 8 clinical pathogenic and 2 non-pathogenic laboratory strains of *E. coli*. Both pathogenic and non-pathogenic strains formed biofilms on the polystyrene microplate (Figure 2C). In almost all pathogenic strains, biofilm mass increased approximately 2-fold between 24 and 48 h.

We then determined the efficacy of the isolated phages in degrading pre-formed *E. coli* biofilms at 24, 48, and 72 h. The phage treatment significantly decreased biofilm formation in *E. coli* strains, with varying degrees of reduction and irrespective of pathogenicity. Substantial biofilm degradation was observed in almost all the *E. coli* strains at 48 h (Figure 2D).

### 3.3. Molecular Characterization and Genome Sequencing of the Phages

The phage isolation from the sewage water sample resulted in a cocktail of bacteriophages, which were enriched by the crossflow purification method and were lyophilized. The genomic characterisation of the phage cocktail was performed by sequencing (n = 3, biological triplicates). After quality checking and trimming the raw reads, de novo assembly was performed using SPAdes. The assembly of Replicate 1 yielded 117 contigs, and the largest contig size was 134,411 bp. The assembly of Replicate 2 contained 20 contigs, with the largest at 134,433 bp. Assembly of Replicate 3 produced 160 contigs, and the size of the largest contig was 128,188 bp.

Upon initial screening of the 3 genome assemblies with PHASTEST, it was found that Replicate 1 had 4 intact phages, Replicate 2 had 2 intact phages, and Replicate 3 had only one intact phage (Supplementary Table S2). Among them, three phage contigs with a coverage of 30× or more were considered for further analysis.

To further validate these predictions, VIBRANT was used on the genome assemblies (Table 1). Among the predicted high-quality genomes across the 3 assemblies, the largest in each was selected for further analysis. Thus, both approaches consistently identified the same 3 phages across the replicates. Further analysis of the phage regions across the replicates by VIBRANT identified multiple virulent phage regions, including 12 phages in Replicate 1, 4 in Replicate 2, and 9 in Replicate 3, with high confidence. Among the phages whose hosts were predicted, 57.89% were classified as *E. coli* infecting double-stranded DNA-tailed phages within the Caudoviricetes class (Supplementary Table S3).

PHASTEST annotated the predicted phage regions. The phages were also manually annotated using Prokka, HMMER, and eggNOG-mapper. Regarding genome size, they varied from 128,188 bp to 134,430 bp. The GC content ranged from 43.68% to 43.76%. The predicted number of CDS ranged from 200 to 212 (Table 2).

**Figure 3:**
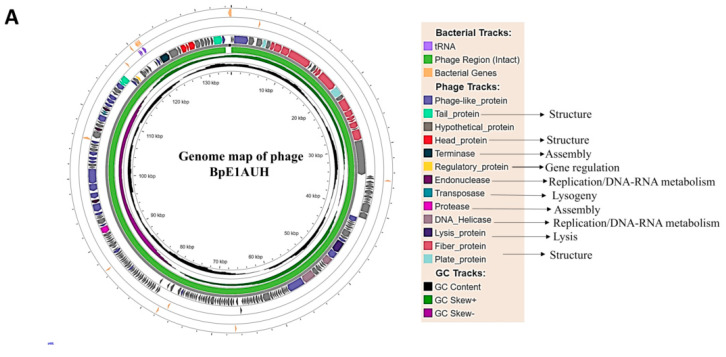

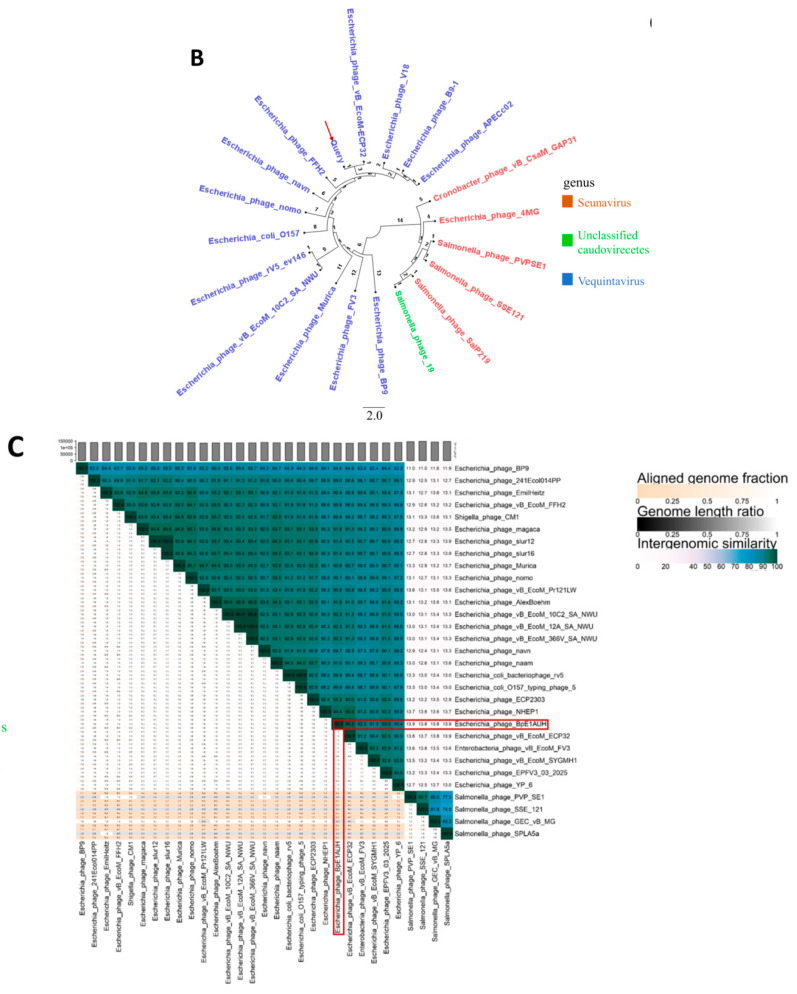

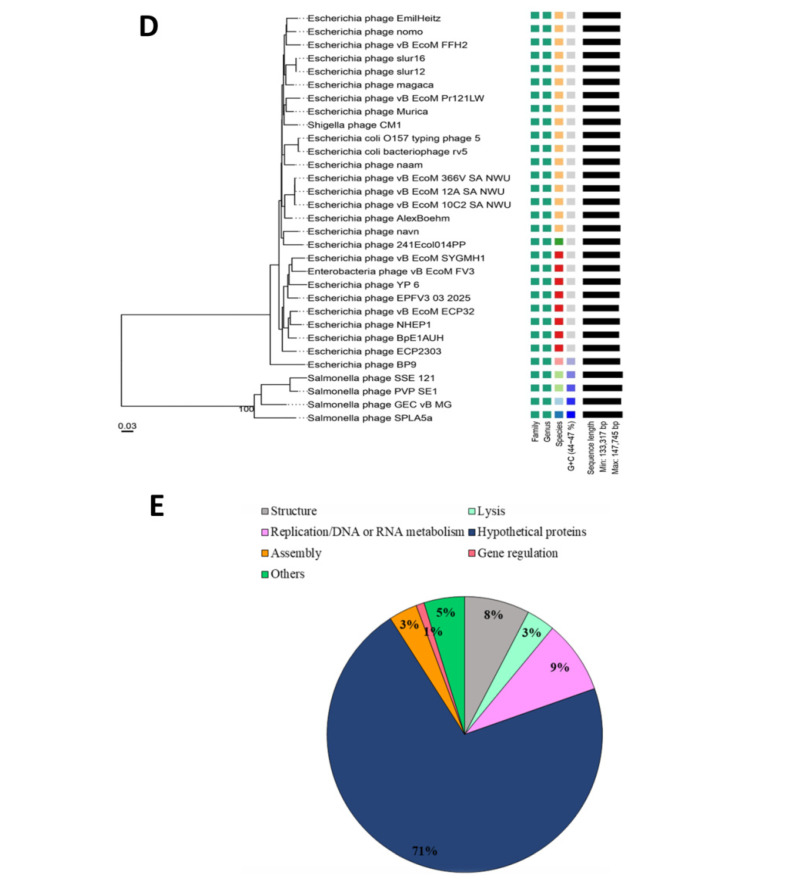
**Whole genome sequencing and characterization of dominant phage (BpE1AUH).** (**A**) Genome map of *Escherichia* phage BpE1AUH. (**B**) Maximum likelihood phylogenetic tree based on the protein sequence of the large terminase subunit of the query phage (indicated by red arrow) and top 20 BLASTp hits. Sequences were aligned using MAFFT v7.505, and the tree was constructed with IQ-TREE. The tree was then visualized with FigTree and displayed in a circular layout. Branch length indicates the number of substitutions per site. The color of the tips suggests the genus: *Seunavirus* (red), Unclassified Caudovirecetes (green), and *Vequintavirus* (blue). The query is placed in the genus *Vequintavirus*, a member of the morphological group Myovirus, along with *Escherichia* phage Murica, facilitating genus-level classification. (**C**) Pairwise Intergenomic similarity of BpE1AUH with 30 related phages, calculated with VIRIDIC. The right half of the map shows the similarity values between phage genomes, while the left half shows the genome length ratio and the fraction of aligned genomes. In the figure, the phage BpE1AUH is highlighted in a red box. (**D**) Phylogenetic GDBP tree based on formula D4. The branch lengths were scaled based on the distance formula, and the tree was rooted at the midpoint. The shapes and colours denote the taxonomic rank of the phages and the G  +  C content. The OPTSIL clustering yielded two family clusters, three genus clusters, and 27 species clusters, which are very diverse, and phage BpE1AUH was predicted as a distinct species. (**E**) Functional classification of phage BpE1AUH CDS into six groups.

The three filtered phages had high sequence similarity based on BLASTn analysis. Phages 1 and 2 had about 99.99% identity with 100% query coverage, whereas Phages 1 and 3 shared 99.99% identity with about 95% query coverage. Among these 3 phages, Phage 1, due to its prevalence, genome completeness, total CDS, and functional genetic profile, was chosen as the representative dominant phage across the three assemblies and analysed in detail in the present study (NCBI GenBank accession ID: PX741096), and designated as *Escherichia* phage BpE1AUH (BpE1AUH). The genome map of BpE1AUH is reported in this study (Figure 3A).

PHASTEST predicted that the phage BpE1AUH might be related to *Escherichia* phage Murica (Murica) (rV5-like) with a nucleotide identity of 93.66% (98% query coverage) based on BLASTn survey, belonging to the class Caudoviricetes and genus *Vequintavirus.* According to the Virus–Host database and eggNOG-mapper results, it belonged to the myovirus morphological group. A BLAST search of the terminase large subunit against the NCBI database further confirmed these results.

CheckV evaluation of the phage BpE1AUH genome revealed it to be of high quality, with an estimated ~100% completeness, direct terminal repeats (DTR), and no contamination. The genome assembly of BpE1AUH was determined to be linear and may follow headful (PAC-type) packaging, with terminal redundancy and partial circular permutation, as inferred by PhageTerm.

### 3.4. Phylogenetic Analysis of the BpE1AUH

To determine the taxonomic position of the phage BpE1AUH, we performed phylogenomic analysis, first with a single gene, followed by the entire phage genome. This resulted in the ML tree, which showed that the terminase large subunit sequence (single-core gene) of the BpE1AUH phage closely clustered with those of other known phages of the genus *Vequintavirus* (*Escherichia* phage vB_EcoM-ECP32 (vB_EcoM-ECP32), Murica, *Escherichia* phage V18, *Escherichia* phage APECc02) (Figure 3B).

We calculated pairwise intergenomic distances using VIRIDIC and found that BpE1AUH shared the highest similarity (96.2%) with vB_EcoM-ECP32 of the *Vequintavirus*, exceeding the threshold (>95%) set by the International Committee on Taxonomy of Viruses (ICTV), suggesting that both belong to the same species. The similarity to phages outside the genus *Vequintavirus* was extremely low (<20%), thus supporting its placement within this genus (Figure 3C).

Further, phylogenetic GDBP tree based on formula D0, D4, and D6 yielded average support of 6%, 21%, and 8%, respectively, indicating moderate diversity of BpE1AUH at the species level with other members. The D0 tree correctly grouped all the *E. coli* and *Salmonella* phages into one single family. But it did not clearly differentiate genus or species, as BpE1AUH was identical to many other phages at the species level (Supplementary Figure S1A). While in D4, there was a clear demarcation, with *E. coli* phages getting clustered under one genus (*Vequintavirus*), while the *Salmonella* phages were grouped into 2 separate clusters at the genus level. Here, BpE1AUH was identified as a distinct species and grouped with ECP32 and NHEP1, although the difference was not significant (Figure 3D). In contrast, in the D6 tree, as in D0, all the phages were grouped into a single genus. But, BpE1AUH, along with 7 other *E. coli* phages, were predicted as the same species (Supplementary Figure S1B).

### 3.5. Annotation

After annotation, more than 70% of the total proteins were classified as hypothetical proteins with unknown functions. To facilitate the curing, the rest 30% of the proteins were grouped into the following categories: virion structure, virion assembly, host lysis, DNA replication/metabolism, gene regulation, and others. Functional categories with a few representatives were grouped under “others,” which included proteins such as metallopeptidase, AAA domain, and phage Ig-like domain, which help phages interact with the bacterial cell surface, etc. (Figure 3E and Supplementary Table S4 (Supplementary Table S4. xlsx)). A tail sheath protein and six lysis proteins were found among them.

## 4. Discussion

Antimicrobial resistance (AMR) is an urgent global crisis, and is estimated to cause more than 39 million deaths globally between 2025 and 2050 [51]. Overuse and abuse of antibiotics are the major culprits that drive AMR in middle-income and low-income countries [52].

Among the many non-traditional antibiotic therapies to tackle AMR, bacteriophages have long been known for their efficacy in limiting bacterial growth [53]. However, identifying potential phages, their purification, their specificity to the bacterial strains, and the lack of regulatory mechanisms hindered the translation of phage therapy into the clinic in India. A recent article by Nagel et al. (2022) indicated that it is more economical and scientifically sound to establish phage banks with locally adapted phages, as they would have coevolved with the regional bacterial strains [13].

The advantage of a phage cocktail is its ability to broaden host-range specificity and suppress phage resistance in hosts by targeting different receptors and host defence systems, such as CRISPR-Cas [54,55]. Furthermore, studies have shown that the phage cocktail can enhance lytic efficiency [54,56]. In agreement with this, our results demonstrate the phage cocktail’s host-range specificity (across different MDR clinical *E. coli* strains) and biofilm degradation efficacy.

In the present study, we have isolated, purified, evaluated, and characterized the bacteriophages present in the sewage water of a Primary Health Centre against pathogenic *E. coli*. Large-scale purification and lyophilization in single-dose vials did not compromise phage efficacy, pH, or temperature stability [57]. Our current study revealed phage tolerance to a wide range of temperatures, from 4 °C to 46 °C, with greater stability at 25 °C, while no activity was detected beyond 60.9 °C. Consistent with previous reports on *E. coli* phages collected from sewage, these observations suggest that phage stability is maintained at moderate temperatures, enabling better storage and handling [58].

The specific pH values were chosen in the current study as they represent physiologically relevant conditions (acidic, basic, and neutral) that phages targeting enteric pathogens might encounter during therapeutic application, consistent with previous reports [58]. As reported in many studies, the morphological features detected using transmission electron microscopy indicated that the predominant phage exhibited myovirus-like morphology [59]. Plaque purification was not performed on the phage lysate in the current study prior to sequencing and morphological characterisation. Thus, the observed morphological heterogeneity was likely due to the mixed phage population (cocktail). All the phages showed specificity for *E. coli* and did not affect *S. aureus* strains.

There was considerable variation in biofilm formation among different *E. coli* strains in vitro. Regardless of their pathogenicity, all the *E. coli* strains formed biofilms. As suggested in the previous reports, biofilm formation by *E. coli* depends on its capacity to adhere to surfaces through physicochemical, molecular, and cellular interactions. These interactions are, in turn, facilitated by structures such as type 1 fimbriae, curli, and F-like conjugative pili, under the influence of temperature, pH, aeration conditions, and nutrient availability [60]. However, these conditions usually apply to domesticated *E. coli* strains traditionally used in laboratories like K-12, DH5α (a derivative of the K-12 strain). Additionally, many other factors, including media type, genetic factors, and environmental conditions, contribute to biofilm formation in pathogenic strains [60].

Consistent with previous studies on *E. coli*, which showed that biofilm usually enters the colonization and development stage around 24 h, then shows a linear increase and attains maximum maturity at 48 h, as observed in the current study [61]. Similarly, after maturity, the reduction seen at 72 h may be linked to cell death due to limited nutrient availability, lack of space, hypoxia, and accumulation of metabolites, which might lead to biofilm detachment from the surface when the media is not renewed [50]. Several studies have already demonstrated the effective usage of phages for biofilm reduction [62]. In the current study, we reported the potential of the phages collected from sewage in degrading the biofilms formed by multidrug-resistant clinical *E. coli* strains, incubated for 3 varying periods. As reported in the earlier studies, this degradation could result from the phage’s ability to adhere to the bacterial cell wall, inject its genome, replicate, and lyse the host bacteria, releasing new phage progeny within the biofilm [63]. A substantial biofilm degradation was observed in almost all *E. coli* strains at 48 h, compared to 24 and 78 h, indicating the phage’s potential to degrade the mature biofilm mass more than at earlier or later stages [63].

Many studies have reported that free-floating, metabolically active bacteria are more prone to phage infection than those embedded in the biofilm matrix [64]. Consistent with these observations, the biofilm degradation observed in the present study is probably correlated with the changing dynamics of biofilm formation over time. At 24 h, due to fewer bacterial cells and immature EPS (Extracellular polymeric substance), measurable biofilm degradation may be limited. The biofilm attains optimal maturity by 48 h, with a mix of free and adherent cells, and becomes more accessible to phages, allowing them to penetrate and display their activity [65]. At 72 h, dense biofilm may make it difficult for phage penetration, and limited nutrition, oxygen, and space in the interior of the biofilm may reduce bacterial susceptibility to phage infection [65]. Our observations are in agreement with the reports that suggested that a phage cocktail is a more suitable option for treating the biofilm of *E. coli* [66].

Genomic analysis of sequenced phage lysate replicates with several in silico tools, such as PHASTEST screening, identified a dominant phage (BpE1AUH) owing to its consistent presence across all replicates and its larger genome size, which was selected for detailed characterisation. PhaBox analysis of phage regions across the three replicates confirmed that the initial lysate represented a mixed phage population, from which phage BpE1AUH was selected. PHASTEST predicted that BpE1AUH might be an *Escherichia* phage Murica (Murica) based on the presence of the tail sheath protein (TSP) [67]. Murica is a lytic phage within the genus *Vequintavirus* that infects Enterotoxigenic *E. coli* (ETEC), responsible for intestinal infections and diarrhoea [68].

VIBRANT analysis further supported this by predicting it as a high-quality lytic phage within the *Vequintavirus* genus. Although VIBRANT predicted it as a circular genome at the assembly level, it usually reflects genome completeness and terminal redundancy rather than the genome’s physical topology. This observation is consistent with PhageTerm’s prediction of a linear genome with partial circular permutation, a feature typically observed in linear headful-packaged dsDNA-tailed bacteriophages (Caudoviricetes) [69].

The CheckV output clearly indicated that BpE1AUH is nearly a complete genome, free of bacterial contamination, and with DTR. As reported earlier, such DTRs found in large linear myoviruses aid in headful packaging and confer terminal redundancy to the phage genome, consistent with the above genomic observations [69].

The functional annotation of the BpE1AUH genome identified four lysis proteins, including a phage lysozyme, an endolysin from the N-acetyl muramidase family [70,71]. As evidenced by previous studies, lysozymes are encoded during the late phase of the phage infection cycle to facilitate host cell lysis and the release of phage progeny by degrading the peptidoglycan layer [70,71]. Previous studies established that biofilm reduction often involves both matrix disruption and bacterial cell lysis [63]. The presence of lysozyme in our phages may help reduce biofilm mass by lysing host cells [63,70].

Additionally, a cell wall hydrolase was observed, along with anti-spanin, a component of the spanin complex required for bacterial outer membrane lysis, as reported earlier [72]. Apart from proteins directly involved in lysis, we also found rIIA and rIIB proteins, usually associated with lytic phages, for regulating the phage DNA replication and lysis process [73,74]. As observed in previous reports, the absence of antibiotic-resistant genes, anti-CRISPR genes, and integrase genes in the phage genome reinforced their lytic behaviour and potential therapeutic usage, specifically for treating infections caused by antibiotic-resistant bacteria [9,75].

Despite the initial prediction of BpE1AUH by PHASTEST as *Escherichia* phage Murica, intergenomic similarity showed the phage’s proximity to vB_EcoM-ECP32, and other related phages still within the *Vequintavirus* genus. Among the phylogenetic GBDP trees made using D0, D4, and D6, the D4 tree was considered more suitable for our phage, as it provided better discrimination at the genus and species levels, placing the BpE1AUH phage as a distinct species, closely related to vB_EcoM-ECP32 and *Escherichia* phage NHEP1. BpE1AUH was grouped together with other *E. coli* phages of the *Vequintavirus* genus in all the trees. The genome size of the phage BpE1AUH (134 kb) is comparable to the typical genome size reported for the members of *Vequintavirus*, 131–140 kb [76]. All these observations strongly support the placement of the phage within the *Vequintavirus* genus. As reported previously, *Escherichia coli* strains are the primary hosts of these phages, consistent with our results [76]. Most of the studies on *Vequintavirus* phages are primarily focused on taxonomic classification and basic characterisation; our present study additionally demonstrated their potential role in biofilm degradation and, therefore, as a possible therapeutic option against pathogenic *E. coli* infections [77]. Accordingly, we deposited the genome of this phage BpE1AUH in NCBI BankIt to enable future taxonomic evaluation and comparative genome studies. (NCBI accession ID: PX741096).

In conclusion, we isolated a cocktail of bacteriophages from sewage water, demonstrated their lytic activity against multidrug-resistant clinical pathogenic *E. coli* strains, and purified them on a large scale. The isolated phage cocktail was specific to *E. coli* and inhibited their biofilm formation, demonstrating effective biofilm degradation. While morphological characterisation revealed heterogeneity, the dominant phage in the cocktail showed myovirus morphology. The in silico prediction and the phylogenetic tree suggested that this predominant phage was a complete phage within the *Vequintavirus* genus, with *E. coli* as its primary host. The genome of this phage was fully characterised, confirming the presence of lytic genes and the absence of antibiotic-resistance genes or an integrase, strongly supporting its lytic nature and potential therapeutic use.

Despite the resources, India still lacks a reference phage bank; therefore, ongoing efforts are required to establish and maintain local phage banks against clinical bacterial strains to reduce the gap between laboratory research and therapeutics.

By demonstrating the physical and genomic stability, lytic activity, specificity for local host–pathogenic bacterial strains, and biofilm-degrading potential of the isolated phage cocktail, our study supports the establishment of phage banks in India.

## Figures and Tables

**Table 1:** Summary of VIBRANT analysis of phage genomes across three replicates.

Replicates of Phage Lysate	No. of Phages Predicted	No. of High-Quality Phages	Predicted Lifestyle	Outline Style		Largest Phage Among High-Quality Phages	
				Linear	Circular	No. of Phages	Length
Replicate 1	17	4	Lytic	3	1	1 (circular)	~134 kb
Replicate 2	4	2	Lytic	1	1	1 (circular)	~134 kb
Replicate 3	27	1	Lytic	1	0	1	~128 kb

**Table 2:** Annotation information of the three phages identified in all the phage genomes.

Phage	Size (In Bp)	GC%	No. of CDS Proteins	No. of tRNA	No. of Annotated CDS	No. of Antibiotic-Resistant Genes	Tail Morphology
PHAGE_Escher_Murica_NC_041871 (from replicate1)	134,410	43.68	212	5	57/212	0	Myovirus
PHAGE_Escher_Murica_NC_041871 (from replicate 2)	133,797	43.68%	210	5	58/210	0	Myovirus
PHAGE_Escher_Murica_NC_041871 (from replicate 3)	128,143	43.76%	200	5	58/200	0	Myovirus

## Data Availability

The data supporting the findings of this study are deposited in the NCBI GenBank database (NCBI accession ID: PX741096).

## Supplementary Materials

The following supporting information can be downloaded at: https://www.mdpi.com/article/10.3390/v18050532/s1. Table S1. Coding of bacterial strains used in the study. Table S2. Phage regions predicted by the PHASTEST in the replicate genomes. Table S3. PhaBox phage prediction results across the three replicate genomes. The dominant phage, BpE1AUH, is highlighted in yellow due to its consistent presence across all the replicates. Table S4. Annotation table. Figure S1. (A): Phylogenetic GDBP tree based on formula D0. The branch lengths were scaled based on the distance formula used, and the tree was rooted at the midpoint. The OPTSIL clustering yielded seventeen species clusters, one genus cluster, and one family cluster. The shapes and colours denote the predicted taxonomic ranks of the phages and the G + C content. All the phages belong to the same family and genus due to identical shape and colour (green squares) in the family and genus column. There is moderate diversity in the species column, where phage BpE1AUH is indicated as a green square, like in the first two columns. (B): Phylogenetic GDBP tree based on formula D6. The branch lengths were scaled based on the distance formula, and the tree was rooted at the midpoint. The shapes and colours denote the taxonomic ranks of the phages and the G + C content.The OPTSIL clustering yielded one family and one genus clusters with identical shape and colour. While it had 7 species clusters, with BpE1AUH being grouped with 7 other phages.
