# Supplementary figures and images for: Isolation and Genomic Characterization of a Lytic Bacteriophage Against Multidrug-Resistant E. coli
Source: Viruses. 2026 Apr 30;18(5):532. doi: 10.3390/v18050532 (PMC13211470; doi:10.3390/v18050532)

Supplementary Fig. 1A.

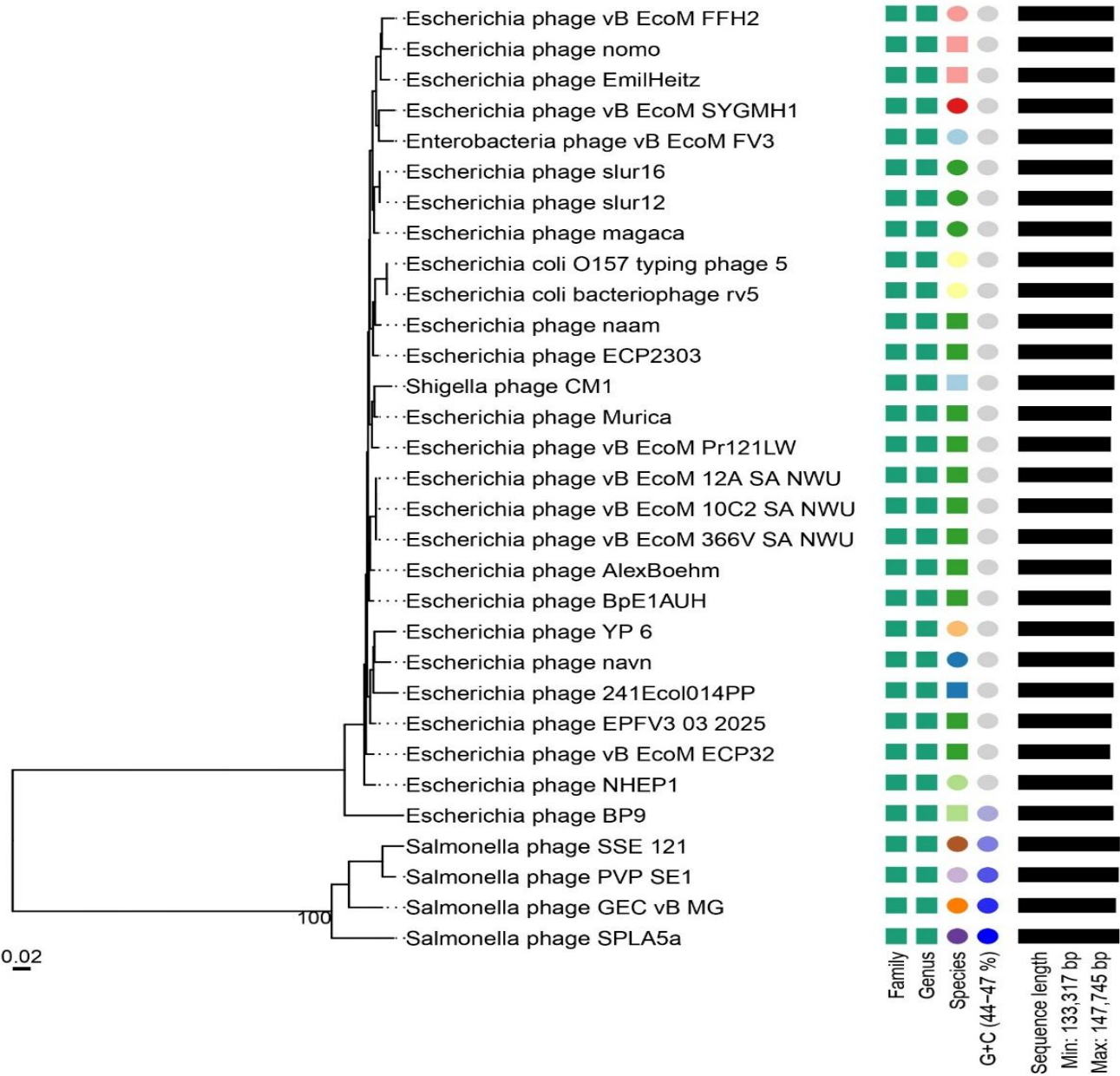

**Supplementary Fig. 1B.**

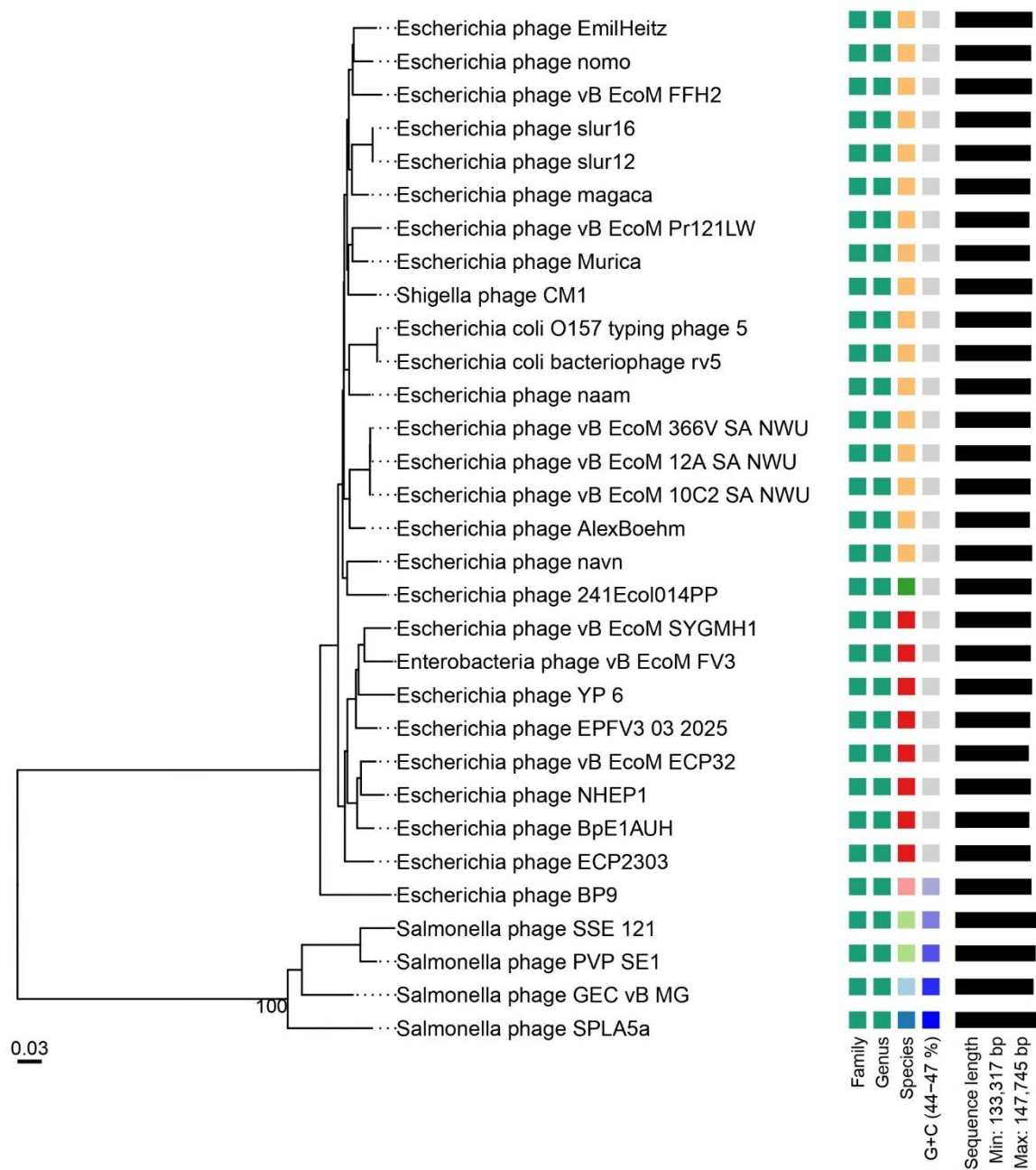

Supplement: Supplementary file 1 [file viruses-18-00532-s001.zip › Supplementary Figure S1.pdf]
